# An Efficient Fabrication Approach for Multi-Cancer Responsive Chemoimmuno Co-Delivery Nanoparticles

**DOI:** 10.3390/pharmaceutics16101246

**Published:** 2024-09-25

**Authors:** Jianxi Huang, Yu-Ting Chien, Qingxin Mu, Miqin Zhang

**Affiliations:** 1 Department of Materials Science and Engineering, University of Washington, Seattle, WA 98195, USA; 2 Department of Pharmaceutics, University of Washington, Seattle, WA 98195, USA

**Keywords:** chemotherapy, immunotherapy, cancer, co-delivery, polymeric nanoparticles

## Abstract

**Background/Objectives:** Cancer remains one of the leading causes of death, with breast, liver, and pancreatic cancers significantly contributing to this burden. Traditional treatments face issues including dose-limiting toxicity, drug resistance, and limited efficacy. Combining therapeutic agents can enhance effectiveness and reduce toxicity, but separate administration often leads to inefficiencies due to differing pharmacokinetics and biodistribution. Co-formulating hydrophobic chemotherapeutics such as paclitaxel (PTX) and hydrophilic immunologic agents such as polyinosinic-polycytidylic acid (Poly IC) is particularly challenging due to their distinct physicochemical properties. This study presents a novel and efficient approach for the co-delivery of PTX and Poly IC using chitosan-based nanoparticles. **Method:** Chitosan-PEG (CP) nanoparticles were developed to encapsulate both PTX and Poly IC, overcoming their differing physicochemical properties and enhancing therapeutic efficacy. **Results**: With an average size of ~100 nm, these nanoparticles facilitate efficient cellular uptake and stability. In vitro results showed that CP-PTX-Poly IC nanoparticles significantly reduced cancer cell viability in breast (4T1), liver (HepG2), and pancreatic (Pan02) cancer types, while also enhancing dendritic cell (DC) maturation. **Conclusions**: This dual-modal delivery system effectively combines chemotherapy and immunotherapy, offering a promising solution for more effective cancer treatment and improved outcomes.

## 1. Introduction

Cancer remains one of the most devastating diseases worldwide, responsible for millions of deaths each year. Among these, breast, liver, and pancreatic cancers significantly contribute to the cancer burden. In 2024, it is estimated that there will be 313,510 new cases of breast cancer in the United States, making it the most common cancer among women [1]. Liver cancer is expected to see about 41,630 new cases, while pancreatic cancer is estimated to result in 66,440 new diagnoses [1]. The treatment of these cancers often involves the single use of chemotherapeutic or immunotherapeutic agents [2,3,4,5,6]. However, conventional cancer treatments face challenges such as severe side effects, drug resistance, and limited effectiveness, leading researchers to explore combinations of different modalities, including chemotherapy and immunotherapy, to enhance therapeutic outcomes [7,8,9].

Chemotherapeutics like paclitaxel (PTX) and immunologic agents like polyinosinic-polycytidylic acid (Poly IC) are widely used in cancer treatment [10]. Chemotherapeutics work by directly killing cancer cells or inhibiting their proliferation [11], while immunologic agents activate the immune system to target and destroy cancer cells [12,13]. Co-delivering these agents can potentially offer synergistic therapeutic benefits by combining their mechanisms of action, enhancing the overall antitumor effect. For instance, Poly IC in combination with doxorubicin has been shown to synergistically kill triple-negative breast cancer cells [14], and Poly IC has been reported to improve the chemotherapeutic efficacy on PTX-resistant colon cancer [15]. Traditionally, chemotherapeutics and immunologic agents are administered separately, which can result in asynchronized and suboptimal pharmacokinetics and biodistributions, as well as increased side effects [16]. A drug carrier capable of delivering multiple agents simultaneously into the tumor’s microenvironment can synchronize the kinetics and translocation of these agents, leading to improved therapeutic efficacy and reduced systemic toxicity.

Nanoparticles have emerged as a promising and the leading solution for the co-delivery of multiple therapeutic and diagnostic agents due to their ability to encapsulate diverse agents, protect the agents from degradation, and enhance their accumulation in tumor tissues through the enhanced permeability and retention (EPR) effect [17]. However, the distinct physicochemical properties of PTX and Poly IC present a significant challenge. The hydrophobic property of PTX and the hydrophilic property of Poly IC make it difficult to co-load them into a single and stable nanoparticle system. In addition, the FDA-approved human serum albumin-bound nanoparticle paclitaxel formulation, Abraxane, showed a negative zeta potential of −26 mV, making these nanoparticles hard to load with negatively charged Poly IC molecules [18]. These limitations highlight the need for an effective nanocarrier system to enhance the delivery and efficacy of these drugs.

Among numerous pharmaceutical excipient materials, chitosan is biocompatible, biodegradable, and has wide range of molecular sizes, making it an excellent base material for nanocarriers. Chitosan is the deacetylated form of chitin, a natural polymer that is second only to cellulose in abundance in the world. Our laboratory has extensively investigated chitosan for various gene and drug delivery purposes [19,20,21,22]. The abundant positively charged amine groups in chitosan facilitate the conjugation of drugs such as PTX and the absorption of negatively charged agents like Poly IC. Chitosan offers significant advantages in protecting a drug from premature degradation, improving drug loading efficiency, and ensuring controlled release [23]. Furthermore, chitosan nanoparticles can easily be modified with polyethylene glycol (PEG) to improve water solubility after paclitaxel attachment, allowing the efficient loading of Poly IC. Additionally, PEG contributed to the formation and maintenance of an optimal hydrodynamic size, crucial for cellular uptake, while also enhancing nanoparticle stability both as a formulation and during systemic circulation, ensuring prolonged in vivo efficacy. Albumin, a protein that occurs naturally in the bloodstream, can further enhance the stability and biocompatibility of these nanoparticles by helping them evade the immune system and prolonging their circulation time [24].

In this study, we present a novel and facile nanoparticle fabrication approach designed for dual modality and capable of multi-cancer treatment (i.e., breast, liver, pancreatic cancers) through the co-delivery of PTX and Poly IC. This straightforward design utilizes chitosan-PEG (CP) as the backbone, which is both biocompatible and effective in delivering therapeutic agents. The nanoparticles were synthesized through ultrasonication and albumin stabilization, producing small, stable particles with an average size of ~100 nm, which is crucial to effective in vivo application. Our approach provides an efficient cancer treatment by leveraging the direct cancer-killing and immune activation effects of PTX and Poly IC within a stable and biocompatible nanoparticle system. This innovative dual-modal strategy offers a promising solution for overcoming single drug resistance, inducing an immune response, and enhancing antitumor immunity in various cancer types.

## 2. Materials and Methods

### 2.1. Materials

All chemicals were purchased from Sigma-Aldrich (St. Louis, MO, USA) unless otherwise stated. Chitosan (MW3900) was purchased from Acmey Industrial Co., Ltd. (Shanghai, China). PTX was purchased from LC Laboratories (Woburn, MA, USA). Bovine serum albumin, wheat germ agglutinin-Alexa Fluor 555 conjugate, 8-well Nunc™ Lab-Tek™ II Chambered Coverglass, eBioscience™ Calcein AM Viability Dye (UltraPure Grade), and propidium iodide were purchased from ThermoFisher Scientific (Waltham, MA, USA). NucBlue DAPI reagent, DMEM, and RPMI 1640 cell culture medium were purchased from Invitrogen (Carlsbad, CA, USA). HyClone characterized fetal bovine serum (FBS) was purchased from GE Healthcare Life Sciences (Pittsburgh, PA, USA). SpectraPOR7 1 kDa RC dialysis tubing was purchased from Repligen Corp (Waltham, MA, USA). The 4T1 and HepG2 cell lines were purchased from the American Type Culture Collection (Manassas, VA, USA). Pan02 cells were purchased from the National Cancer Institute (NCI) Division of Cancer Treatment and Diagnosis (DCTD) Tumor Repository (Frederick, MD, USA). Recombinant Murine GM-CSF was purchased from Peprotech (Cranbury, NJ, USA). The CellTiter-Glo^®^ 3D cell viability assay kit was purchased from Promega (Madison, WI, USA). Fixation buffer, TruStain FcX™ PLUS (anti-mouse CD16/32) antibodies, PE anti-mouse CD11c, APC anti-mouse CD86 antibodies, and FTIC anti-mouse CD80 antibodies were purchased from BioLegend (San Diego, CA, USA).

### 2.2. Synthesis of PTX-COOH

The synthesis of PTX-COOH followed a previously established method with some modifications [25]. Briefly, PTX (50 mg) and succinic anhydride (11.8 mg) were dissolved in 6 mL of chloroform containing 56.9 μL of pyridine. This mixture was stirred at room temperature for 24 h and then dried overnight in a vacuum oven. The resulting dried sample was washed three times with deionized water using centrifugation (4000× *g*, 2 min). The sample was then dissolved in 2 mL of acetone and transferred to a 15 mL conical tube. Deionized water was gradually added to the acetone solution until it turned turbid (approximately 10 mL of water). The suspension was centrifuged for 10 min at 1000× *g*. After removing the supernatant, 2 mL of deionized water was added to resuspend the PTX-COOH. The suspension was then freeze-dried.

### 2.3. Synthesis of CP-PTX-Poly IC and Cy5-Labeled CP-PTX-Poly IC

Chitosan-PEG (CP) was synthesized following a previously established procedure [26]. PTX-COOH (1.1 mg), EDC (0.8 mg), and NHS (0.4 mg) were dissolved in 0.5 mL of DMSO in a microtube and incubated for 3 h on a rocker. CP (20 mg) was dissolved in 0.8 mL of sodium bicarbonate buffer (pH 8.5) in a 20 mL glass vial and diluted with 1.6 mL of DMSO. The PTX-COOH/EDC/NHS DMSO solution was then added into the CP solution under stirring. The mixture was stirred overnight. To reduce the DMSO percentage, 8 mL of deionized water was gradually added to the mixture under continuous stirring. The resulting solution was dialyzed against 2 L of deionized water for 24 h using 1 kDa RC dialysis tubing, with water changes at 1, 3, and 7 h. All samples, including any precipitates, were transferred into a 20 mL glass vial and subjected to sonication for 10 min using a Sonic Dismembrator Model 500 (Fisher Scientific, Pittsburgh, PA) at 40% amplitude, with 10 s on and 5 s off cycles. The solution was then centrifuged at 20,000× *g* for 10 min. The supernatant was collected and stored in the fridge. For the conjugation of Cy5, 0.3 mL 10 × PBS buffer (pH 7.4) was added into 2.7 mL of the CP-PTX solution. Ten (10) μL of NHS-Cy5 (5 mg/mL) was then added into the mixture solution of PBS and CP-PTX, and the resulting solution was incubated on a rocker at room temperature for 2 h. The solution was then dialyzed against deionized water for 24 h, with the water being changed three times using 1 kDa RC dialysis tubing. The concentration of CP-PTX/Cy5-labeled CP-PTX was calculated by measuring the mass of CP-PTX/Cy5-labeled CP-PTX powder after freeze-drying 10 mL of the CP-PTX/Cy5-labeled CP-PTX solution. Poly IC (1 mg/mL) was added to CP-PTX/ Cy5-labeled CsP-PTX solution at specified mass ratios. To stabilize the CP-PTX-Poly IC, albumin was added to CP-PTX-Poly IC solution to reach a final concentration of 0.3 mg/mL of albumin.

### 2.4. ^1^H NMR

All NMR experiments were conducted using a Bruker GG-500 spectrometer (Karlsruhe, Germany) at room temperature with a ^1^H frequency of 499.65 MHz. CP and CP-PTX were dissolved in D2O, while PTX was dissolved in DMSO-d6 for the measurements. The scanning parameters were set to NS = 96, AQ = 2.34 s, and TD = 32,678. For the quantification of PTX in CP-PTX, the dried sample was dissolved in 0.9 mL of D2O and mixed with 3-(Trimethylsilyl)propionic-2,2,3,3-d4 acid sodium salt (TSP) solution (17.2 mg/mL dissolved in 0.1 mL of D2O) before capturing the spectra. Peaks solely from TSP (δ = 0, 9H) and PTX (δ = 7.75, 2H) were integrated. The absolute concentration of PTX in the solution was then calculated using the following equation:

(1) $$m_{D}=\frac{m_{IS}P_{IS}I_{D}M_{D}}{M_{IS}P_{D}I_{IS}}$$

where *m_D_* is the mass of PTX, *m_IS_* is the mass of the internal standard (TSP), *P_D_* and P_IS_ are the numbers of protons responsible for the resulting peak in the drug and the internal standard, respectively; *I_D_* and *I_IS_* are the integration values of the peaks of the drug and the internal standard, respectively; *M_D_* and *M_IS_* represent the molar masses of the drug and the internal standard, respectively.

### 2.5. TEM Imaging

The TEM sample was prepared by adding 5 μL of CP-PTX-Poly IC solution to a formvar/carbon-coated 300-mesh copper grid (Ted Pella, Inc., Redding, CA, USA) and allowed to air dry. The TEM images were acquired on a Tecnai G2 F20 electron microscope (FEI, Hillsboro, OR, USA) operating at a voltage of 200 kV. The size distribution of CP-PTX-Poly IC was analyzed using ImageJ software (version 1.53t).

### 2.6. Hydrodynamic Size, Polydispersity Index (PDI), Zeta Potential, Conductivity Measurement, and Long-Term Stability

The hydrodynamic size, PDI, zeta potential, and conductivity of the CP-PTX-Poly IC samples in Corning^®^ molecular biology grade water or phosphate buffered saline (PBS) (Fisher Scientific, Pittsburgh, PA) were determined using a Zetasizer Nano-ZS (Malvern Instruments, Worcestershire, UK). To investigate the effect of Poly IC on CP-PTX (with/without albumin), measurements were performed in water at room temperature. To study the long-term stability of CP-PTX-Poly IC, samples dissolved in water were stored in a 37 °C water bath for 7 days and the size measurement was conducted at room temperature daily.

### 2.7. Drug Release Study

PTX release was assessed by a dialysis method. CP-PTX-Poly IC solution was loaded into two 3.5 kDa MWCO dialysis bags (1 mL each). The sealed dialysis bags were then immersed in 30 mL of two different buffers (pH 7.4 (PBS + 0.1% Triton X-100) and pH 5.4 (sodium acetate buffer + 0.1% Triton X-100) at 37 °C and stirred for 24 h). The PTX release was sampled (0.5 mL each, then freeze-dried) at different time points (2, 18, 24 h) and quantified by high-performance liquid chromatography (HPLC). The cumulative release was calculated by measuring the PTX concentration in the samples and converting the measurements to show the total percentage of drug released over time.

The PTX was quantified with a Shimadzu HPLC-UV system (Kyoto, Japan). Chromatographic separation was achieved using a KinetexC18 column (100 Å, 5 μm, 4.6 mm × 100 mm) (Phenomenex, Torrance, CA). The flow rate was set to 1.0 mL/min with a 15 μL sample injection volume. The mobile phase for separation consisted of pumps A (Acetonitrile) and B (10 mM Ammonium Acetate in water). The gradient program used was as follows: pump B was set to 40% and increased to 100% over 5 min. The wavelength for the detection of PTX was 254 nm.

### 2.8. Cell Culture

The 4T1 and Pan02 cells were cultured in RPMI1640 medium supplemented with 10% vol/vol FBS and 1% vol/vol penicillin-streptomycin (Pen strep). HepG2 cells were cultured in DMEM medium supplemented with 10% vol/vol FBS and 1% vol/vol penicillin-streptomycin. Cultures were maintained in a 37 °C, 5% CO_2_ humidified incubator.

### 2.9. Cellular Uptake Study

The 4T1, HepG2, and Pan02 cells were seeded at 8,000, 16,000 and 8,000 cells per well, respectively, on an 8-well chambered cover glass and incubated for 24 h before treatment. Cells were untreated or treated with Cy5-labeled CP-PTX-Poly IC at 100 μg/mL for 2 h in a 37 °C incubator. Cells were then washed with cold PBS twice and fixed with a fixation buffer (4% paraformaldehyde in PBS) for 10 min. The fixed cells were then washed with cold PBS twice before their membranes were stained by WGA-Alexa Fluor 555 for 5 min. Next, 100 μL of NucBlue FixCell ReadyProbe DAPI reagent (diluted 10 times in cold PBS) was added to each well after the stained cells were washed again with cold PBS two more times. Confocal images were acquired using a Leica SP8X confocal laser scanning microscope (Leica, Wetzlar, Germany).

### 2.10. Cancer Cell Viability Study

The 4T1, HepG2, and Pan02 cells were seeded at 4,000, 8,000 and 4,000 cells per well, respectively, in 96-well plates and incubated for 24 h before treatment. The cells were treated with different agents (Poly IC, CP, PTX, CP-PTX, or CP-PTX-Poly IC) for 48 h. The dose of each agent was equivalent to the corresponding component amount in CP-PTX-Poly IC at 2 µM PTX (Poly IC = 425 μg/mL). The PTX amounts of CP-PTX and CP-PTX-Poly IC were determined via NMR analysis. Brightfield images were acquired using a Nikon TE300 inverted fluorescent microscope (Nikon, Tokyo, Japan). The relative ATP level was measured using a CellTiter-Glo^®^ 3D cell viability assay kit following the manufacturer’s protocol. The relative ATP levels of all of the treatment groups were normalized so that the ATP level of the untreated cell group was 100. Live/dead cell staining was performed on both the untreated cells and the PTX-treated cells. The cells were stained with calcein AM and propidium iodide according to the manufacturer’s protocol. The stained cells were then imaged, and their live cell intensities were calculated using ImageJ software (version 1.53t).

### 2.11. Bone Marrow-Derived Dendritic Cells (BMDCs) Harvest and Culture

BMDCs were harvested from female C3(1)-tag mice of 6–10 weeks old in accordance with University of Washington Institute of Animal Care and Use Committee (IACUC) approved protocols (Identification code: 3441-05, approval date: 4/14/2022) as well as with federal guidelines. The isolation of bone marrow cells was carried out following a BMDC isolation protocol [27]. The cells were resuspended in RPMI-1640 supplemented with 10% FBS, 20 ng/mL recombinant Murine GM-CSF, and 1% Pen Strep. The cells were allowed to differentiate for 7 days with fresh media added on day 3. On day 7, the cells were aspirated and counted for assays. A medium containing 10 ng/mL GM-CSF was used in the assays.

### 2.12. BMDC Activation and Cytotoxicity Study

BMDCs were seeded at 30,000 cells per well in a 96-well plate. The cells were treated using different agents (Poly IC, CP-PTX, or CP-PTX-Poly IC). These agents were free of foreign particles and dissolved in sterile biology grade water. The dose of each agent was equivalent to the corresponding component amount in CP-PTX-Poly IC at 10 μg/mL Poly IC for 24 h. Untreated cells were used as a reference. In the BMDC activation study, the cells were dissociated from the plate using Trypsin-EDTA (0.05%) (ThermoFisher, USA) and washed twice in cold PBS. TruStain FcX™ PLUS (anti-mouse CD16/32) antibodies were used to block the Fc-receptors of the cells for 30 min to prevent non-specific immunofluorescent staining. The blocked cells were then washed in cold PBS twice before being stained with PE-CD11c (0.2 mg/mL), APC-CD80, and FITC-CD86 antibodies (5 µg/mL each antibody) for 20 min. The stained cells were washed with PBS twice. The CD80 and CD86 expression levels in the CD11c+ cells were analyzed using a flow cytometer. For the BMDC cytotoxicity study, brightfield images were acquired using a Nikon TE300 inverted fluorescent microscope. The relative ATP level was measured using a CellTiter-Glo^®^ 3D cell viability assay kit following the manufacturer’s protocol. The relative ATP levels of all of the treatment groups were normalized so that the ATP level of the untreated cell group was 100.

### 2.13. Statistical Analysis

The results were presented as mean values ± standard error of the mean. The statistical differences were determined by one-way ANOVA with post hoc tests.

## 3. Results and Discussion

### 3.1. Design of CP-PTX-Poly IC Nanoparticle

**Figure 1:**
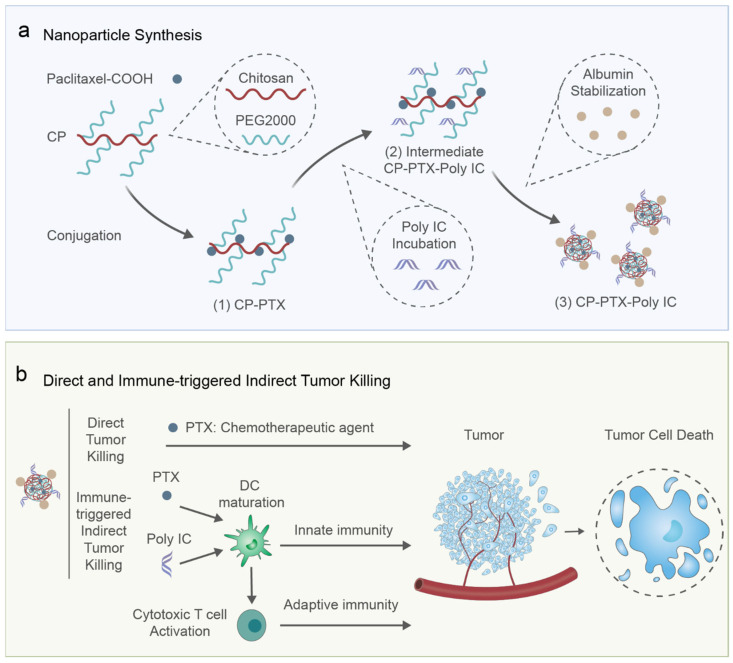
CP-PTX-Poly IC nanoparticle that co-delivers PTX and Poly IC to cancer cells and dendritic cells for combined chemo-immunotherapy. (**a**) Schematic illustration of nanoparticle synthesis. PTX was covalently conjugated on CP polymers that contain chitosan and PEG, forming CP-PTX (intermediate (1)). The CP-PTX was incubated with Poly IC (intermediate (2)) before albumin stabilization into CP-PTX-Poly IC nanoparticles (product (3)). (**b**) Potential mechanisms of combined chemo-immunotherapy for the co-delivery of PTX and Poly IC for direct tumor killing and immune-triggered indirect tumor killing in vivo. The latter includes the activation of the host’s immune system by NPs through dendritic cell (DC) maturation and the subsequent activation of anti-cancer innate and adaptive (activation of cytotoxic T cells) immune responses.

We designed a nanoparticle with dual antitumor modalities, combining the chemotherapeutic agent paclitaxel (PTX) and the immunotherapeutic agent polyinosinic-polycytidylic acid (Poly IC) (Figure 1a). The synthesis process involved several key steps. First, PTX was carboxylated to form PTX-COOH, which was then covalently attached to a biocompatible and hydrophilic polymeric carrier composed of chitosan and PEG (CP). The synthesis of CP followed a previously established procedure [26], in which the aldehyde activated methoxy-PEG reacted with the amine groups of chitosan in the presence of sodium cyanoborohydride as a reducing agent, forming a stable covalent bond between chitosan and PEG. As both chitosan and PEG are highly hydrophilic, the conjugation of relatively small amounts of hydrophobic PTX resulted in water soluble CP-PTX intermediate (Figure 1a, intermediate (1)). Due to the abundant primary amine groups on chitosan, the CP-PTX carried a positive charge. Next, the negatively charged Poly IC was electrostatically bound to the CP-PTX, forming intermediate CP-PTX-Poly IC (Figure 1a, intermediate (2)). To enhance stability and suitability for systemic administration, albumin was electrostatically absorbed onto the surface of the CP-PTX-Poly IC via co-incubation, creating stabilized CP-PTX-Poly IC nanoparticles (Figure 1a, product (3)). The addition of albumin offers potential benefits of enhancing tumor uptake, as it can interact with the glycoprotein 60 (GP60) and secreted protein acidic and rich in cysteine (SPARC) receptors found on vascular endothelial cells and tumor cells, respectively [28].

Figure 1b illustrates the potential interactions of these nanoparticles within the tumor microenvironment. Once internalized by tumor cells, the acidic conditions in endosomes or lysosomes trigger the release of PTX via the hydrolysis of the amide bond between the PTX and the carrier, leading to direct tumor cell apoptosis. Additionally, the PTX and Poly IC on the nanoparticles can interact with tumor-infiltrating dendritic cells (DCs) and be internalized, thereby activating an antitumor immune response. As key antigen-presenting cells, DCs play a crucial role in initiating and regulating immune responses. Early DC maturation can be enhanced by PTX due to its ability to signal through toll-like receptor 4 (TLR4) on DC cells [29]. In addition, the binding of TLR3 expressed on DCs with Poly IC stimulates DC maturation and induces the expression of co-stimulatory molecules such as CD80 and CD86 [30]. Mature DCs also activate cytotoxic natural killer (NK) cells and T cells, leading to innate and adaptive immunity against tumors [31,32]. Consequently, the designed nanoparticles can interact with both tumor cells and the immune system following systemic administration, trigger direct and indirect cancer-killing mechanisms in vivo, and achieve improved therapeutic outcomes compared to conventional single-agent treatments.

### 3.2. Synthesis and Structural Validation of CP-PTX

**Figure 2:**
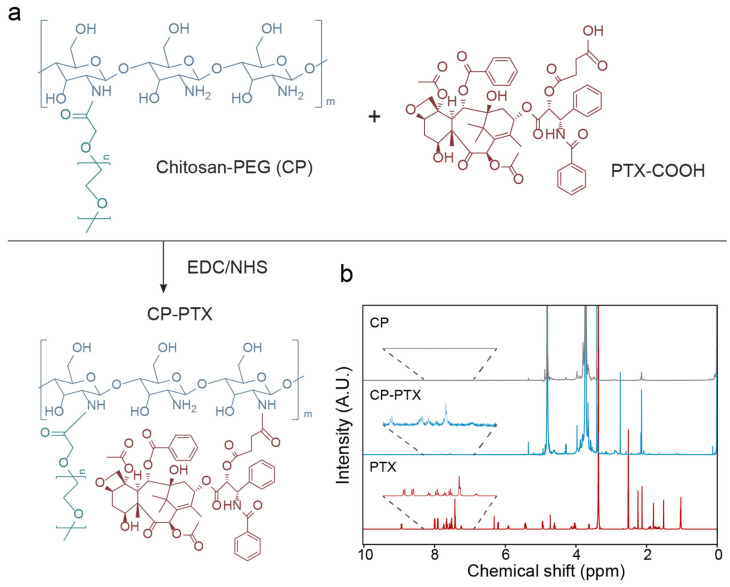
Synthesis of CP-PTX. (**a**) Schematic representation of CP-PTX synthesis from CP and carboxylated PTX via EDC/NHS chemistry. (**b**) NMR spectra of CP, CP-PTX, and PTX.

We synthesized CP-PTX by conjugating carboxylated paclitaxel (PTX-COOH) to a chitosan-PEG (CP) carrier, producing a stable and soluble nanoparticle for drug delivery (Figure 2a). Low molecular weight chitosan (MW = 3900) was modified with PEG to enhance its solubility and maintain stability after hydrophobic drug conjugations. PTX was carboxylated to form 2′-succinyl PTX (PTX-COOH) using succinic anhydride under basic conditions, with the expectation that PTX would be released into the acidic and enzymatic tumor microenvironment [33]. PTX-COOH was then covalently bonded to the primary amines on CP through an EDC/NHS reaction in a sodium bicarbonate (pH 8.5)/dimethyl sulfoxide (DMSO) solution, forming CP-PTX.

The covalent bonding between PTX and CP was confirmed using nuclear magnetic resonance (NMR) spectroscopy (Figure 2b). The NMR spectra of CP-PTX showed characteristic peaks of chitosan and PEG at 3.5–4 ppm, along with the aromatic protons of PTX at 7–8.2 ppm, indicating successful conjugation. In contrast, these aromatic protons were absent in the spectra of CP alone. In our previous work, when CP and PTX were conjugated using the same reaction scheme, the formation of an amide bond between the two agents was confirmed through Fourier transform infrared spectroscopy (FTIR) analysis [25]. The PTX loading on CP-PTX was quantitatively determined to be up to ~4.75 wt% using the NMR method. The release of PTX from CP-PTX nanoparticles was assessed using HPLC by analyzing the dialysate under two pH conditions, simulating physiological pH (7.4) and the acidic environment of cellular endosomes (5.4). A clear pH-dependent release profile was observed (Figure S1). After 24 h, only 34.3% of the loaded PTX was released at pH 7.4, whereas a significantly higher release of approximately 60.9% was observed at pH 5.4. These findings indicate that PTX remains largely retained in the nanoparticles in the bloodstream but is efficiently released once internalized by target cells and exposed to the acidic conditions of the lysosomes.

### 3.3. Physiochemical Properties of CP-PTX-Poly IC

**Figure 3:**
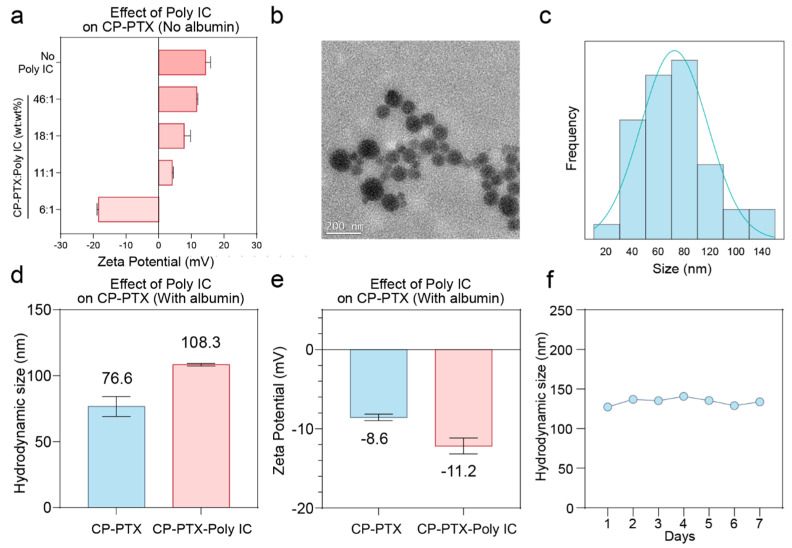
Physicochemical properties of CP-PTX-Poly IC nanoparticles. (**a**) Zeta potentials of CP-PTX (intermediate (1) in Figure 1a) and a series of intermediate CP-PTX-Poly IC complexes (intermediate (2) in Figure 1a) at different CP-PTX: Poly IC ratios (wt: wt% = 46:1, 18:1, 11:1, 6:1) at room temperature. (**b**) TEM micrographs of final CP-PTX-poly IC nanoparticles. Scale bar is 200 nm. (**c**) Size distribution of CP-PTX-Poly IC nanoparticles analyzed from TEM images. (**d**) Hydrodynamic size and (**e**) zeta potential of albumin-stabilized CP-PTX and CP-PTX-Poly IC nanoparticles (step (3) in Figure 1a). Measurements were conducted at room temperature in water (pH ~7). (**f**) Hydrodynamic size of CP-PTX-Poly IC at 37 °C in water over 7 days.

The physiochemical properties of CP-PTX-Poly IC were further analyzed. Negatively charged Poly IC was absorbed onto CP-PTX through electrostatic interactions. To optimize Poly IC loading, we evaluated the zeta potentials of CP-PTX-Poly IC at various CP-PTX: Poly IC mass ratios (Figure 3a). CP-PTX without Poly IC exhibited a zeta potential of +14.3 mV. With increasing amounts of Poly IC, the zeta potential decreased to +11.6, +7.8, and +4.1 mV for CP-PTX-Poly IC ratios of 46:1, 18:1, and 11:1, respectively, indicating the successful loading of Poly IC. When Poly IC was in excess, the zeta potential turned negative, reaching −18.4 mV at a CP-PTX-Poly IC ratio of 6:1. For further stabilization with net negatively charged albumin (at pH 7), the intermediate CP-PTX-Poly IC should retain a positive charge. Therefore, we selected the 11:1 CP-PTX-Poy IC ratio as the optimal formulation, balancing maximal Poly IC loading with a positive charge. The selected formulation had a PTX: Poly IC mass ratio of 1:14 and served as a proof-of-concept for the co-delivery system. The drug ratio can be tailored according to clinical applications. The intermediate CP-PTX-Poly IC at the selected formulation was then incubated with albumin, forming stabilized CP-PTX-Poly IC nanoparticles for subsequent studies.

Nanoparticle size is crucial for delivery applications, impacting stability in the bloodstream and cellular interactions. Nanoparticles larger than 200 nm can activate the complement system, leading to rapid clearance and accumulation in the liver and spleen. Conversely, nanoparticles smaller than 10 nm are quickly eliminated by the kidneys [34]. Achieving an optimal size range ensures prolonged circulation and effective delivery. The morphology of the CP-PTX-Poly IC nanoparticles was characterized by transmission electron microscopy (TEM) (Figure 3b). TEM images revealed homogenous nanoparticles with an average diameter of 72.2 nm, analyzed using ImageJ software (Figure 3c). The hydrodynamic size in water, measured by dynamic light scattering (DLS), was 108.3 nm, slightly larger due to the hydration of the PEG and the association of water molecules around the nanoparticles (Figure 3d). In the physiological environment, the hydrodynamic size slightly increased due to reduced electrostatic interaction, which was caused by the presence of the salts (i.e., Na^+^ and Cl^−)^, as evidenced by the hydrodynamic size of 141.1 nm tested in the PBS solution (Figure S2). The small size of CP-PTX-Poly IC not only facilitates the cell internalization of these nanoparticles but could also be favorable for long-term in vivo trafficking.

We conducted zeta potential measurements to compare albumin-stabilized CP-PTX (no Poly IC) and CP-PTX-Poly IC (Figure 3e). The zeta potentials are −8.6 and −11.2 mV for CP-PTX and CP-PTX-Poly IC, respectively. Since the protein-stabilized CP-PTX-Poly IC was larger and more negatively charged than the stabilized CP-PTX, the successful loading of Poly IC on CP-PTX-Poly IC was further confirmed.

Stability is another critical factor for nanoparticle-based drug delivery systems. We assessed the stability of the CP-PTX-Poly IC nanoparticles by monitoring their hydrodynamic size in water at 37 °C over seven days. The nanoparticles maintained a consistent size range of 127–140 nm, indicating good stability under physiological conditions (Figure 3f). This stability is essential for ensuring that the nanoparticles remain intact and functional during circulation before delivery to tumor sites.

### 3.4. Cellular Uptake of CP-PTX-Poly IC Nanoparticles

To ensure the effective delivery of the drugs to the cancer cells, we investigated the cellular uptake of CP-PTX-Poly IC in three different cancer cell lines: breast cancer cell line 4T1, liver cancer cell line HepG2, and pancreatic cancer cell line Pan02. The 4T1 cells are murine breast cancer cells that are often used as a model of human metastatic triple-negative breast cancer, characterized by the lack of an estrogen receptor (ER), progesterone receptor (PR), and HER2 expression [35]. HepG2 cells are derived from human liver carcinoma and are commonly used to study liver cancer biology and drug metabolism [36]. Pan02 cells are derived from the pancreatic ductal tissue of C57BL/6 mice and exhibit high proliferation, invasiveness, and metastatic potential, making them a suitable model for studying pancreatic cancer progression and therapy [37].

**Figure 4:**
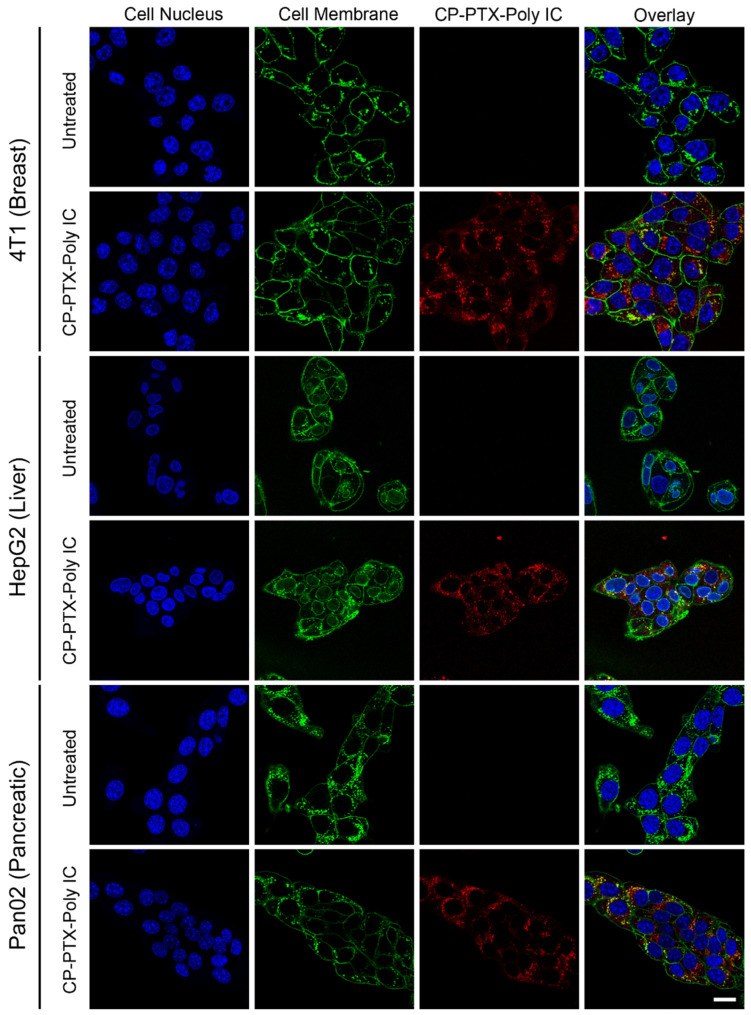
Cellular uptake of CP-PTX-Poly IC NPs in three cancer types. Confocal images of 4T1, HepG2, and Pan02 cells incubated for 2 h with Cy5-labeled CP-PTX-Poly IC. Untreated cells were used as a reference. Blue color represents cell nucleus and green color represents cell membrane. Red color is the signal of CP-PTX-Poly IC. Scale bar is 20 μm.

To visualize and confirm the intracellular localization of the nanoparticles, we synthesized labeled CP-PTX-Poly IC by conjugating CP-PTX with Cy5 fluorophores before incubating with Poly IC and albumin. The three cell lines were treated with Cy5-labeled CP-PTX-Poly IC for 2 h before the nuclei were stained with DAPI (blue) and their plasma membranes were stained with WGA-AF555 (green). Confocal microscopy images (Figure 4) revealed that a substantial amount of CP-PTX-Poly IC penetrated the plasma membrane and localized within the intracellular spaces of 4T1, HepG2, and Pan02 cells (Figure 4, red color). This indicates that the cellular internalization process of CP-PTX-Poly IC was efficient across all three cancer cell lines, independent of the site of a cancer’s origin and its molecular profile.

The efficient cellular uptake of CP-PTX-Poly IC is critical for ensuring that the therapeutic agents reach their intracellular targets. The successful internalization of these nanoparticles suggests their potential effectiveness in delivering chemotherapeutic and immunotherapeutic agents directly to cancer cells, thereby enhancing the overall therapeutic outcomes.

### 3.5. Cancer Cell Viability Study

The cancer-killing effect of CP-PTX-Poly IC nanoparticles was evaluated in three cancer cell lines: 4T1, HepG2, and Pan02. Each cell line was treated with different formulations, including Poly IC, CP, PTX, CP-PTX, and CP-PTX-Poly IC, all administered at the corresponding component amounts equivalent to CP-PTX-Poly IC at 2 μM PTX. The treatments were applied for 48 h.

**Figure 5:**
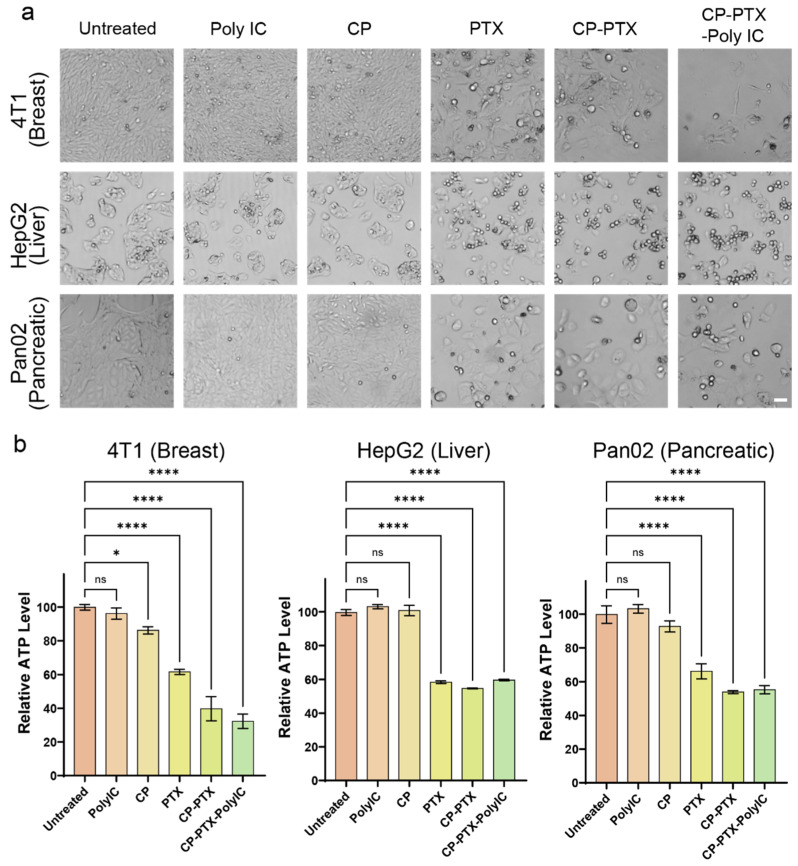
Cancer killing effect of CP-PTX-Poly IC. (**a**) Brightfield images and (**b**) Relative ATP level of 4T1 (Breast), HepG2 (Liver), and Pan02 (Pancreatic) cells treated with Poly IC, CP, PTX, CP-PTX, and CP-PTX-Poly IC for 48 h. Scale bar is 50 μm. The dose of each treatment was the same amount of the corresponding components in CP-PTX-Poly IC at 2 µM PTX (Poly IC = 425 μg/mL). * *p* < 0.05, **** *p* < 0.0001, ns = statistically insignificant.

Brightfield images of the untreated and treated cells were collected (Figure 5a). Poly IC and CP-treated cells showed minimal changes in terms of cell number and morphology compared to the untreated cells in three cell lines. In contrast, a remarkable reduction of cell number was seen in cells treated with PTX-containing groups. Notably, 4T1 cells treated with CP-PTX-Poly IC were mostly killed. In HepG2 cells, a considerable amount of apoptosis cells was observed in the CP-PTX-Poly IC treatment group. Pan02 cells treated with CP-PTX-Poly IC showed not only apoptosis, but also cell enlargement, due to the disruption of cell division. This effect is consistent with the known mechanism of PTX, which binds to the β-tubulin subunit of microtubules, the structural components essential for cell division, disrupting mitosis and leading to cell death [38]. The observed variation in anti-cancer activities between the three cell types might be due to inherent biological differences. Known for their rapid proliferation and high metastatic potential, 4T1 cells might be more sensitive to the combined chemo-immunotherapeutic effect of PTX and Poly IC because these drugs target rapidly dividing cells more effectively [39].

To quantitively evaluate the cells’ viability, their adenosine triphosphate (ATP) levels were assessed as indicators of their viability in all the treatment groups using a CellTiter-Glo^®^ 3D cell viability assay kit (Figure 5b). The ATP levels of the treated cells were normalized to the ATP level of the untreated control cells, which was set to 100. Results showed a consistent trend (as shown in the brightfield image results) in which Poly IC and CP exhibited minimal cytotoxicity while PTX, CP-PTX, and CP-PTX-Poly IC demonstrated significant cancer-killing effects. In 4T1 cells, the relative ATP level of cells treated with Poly IC was 96.2 while that of cells treated with CP was slightly lower at 86.2. Free PTX showed a relative ATP level of 61.6. The ATP levels of the cells treated with CP-PTX and CP-PTX-Poly IC were even lower, at 39.7 and 32.3, respectively. The trend in two other cell lines, HepG2 and Pan02, was similar, but the ATP levels of PTX, CP-PTX, and CP-PTX-Poly IC were not clearly distinguished. CP-PTX-Poly IC led to ATP levels of 59.7 and 55.2 in HepG2 and Pan02, respectively. Since ATP levels serve as an indirect indicator of cell viability, a live/dead cell staining was performed to verify the accuracy of the assay. Compared to the untreated live cell intensities, set at 100, the live cell intensities of PTX-treated 4T1, HepG2, and Pan02 cells were measured at 53.3, 53.8, and 51.8, respectively. These values were slightly lower than the corresponding ATP levels of 61.6, 58.4, and 66.2, indicating that the ATP measurement accounts for the majority of live cells but also includes some dead cells. Therefore, the actual cancer-killing effect may be more significant than the ATP levels suggest, highlighting the potent cytotoxicity of CP-PTX-Poly IC.

Overall, the CP-PTX-Poly IC nanoparticles demonstrated significant cytotoxicity in breast, liver, and pancreatic cancer cell lines, primarily due to the action of PTX. The co-delivery of PTX and Poly IC to cancer cells can not only induce cell apoptosis, but also promote cellular production of cytokines and chemokines. In a study conducted by Du et al., it has been shown that the co-delivery of PTX and Poly IC to murine melanoma cells activated cellular production of cytokines and chemokines, including IFN-β, IL-6, CCL-5, and CXCL-10 in vitro. These secreted immune signaling molecules stimulated immune cells, leading to elevated cancer-killing effects [40].

### 3.6. In Vitro Immune Response of Bone Marrow-Derived Dendritic Cells (BMDCs) to CP-PTX-Poly IC

To investigate the activation and viability of dendritic cells in response to different treatments, bone marrow-derived dendritic cells (BMDCs) were treated with various agents, including Poly IC, CP-PTX, and CP-PTX-Poly IC for 24 h. The dose of each agent was the same as the corresponding component in CP-PTX-Poly IC at 10 μg/mL of Poly IC. Untreated cells were used as a control.

**Figure 6:**
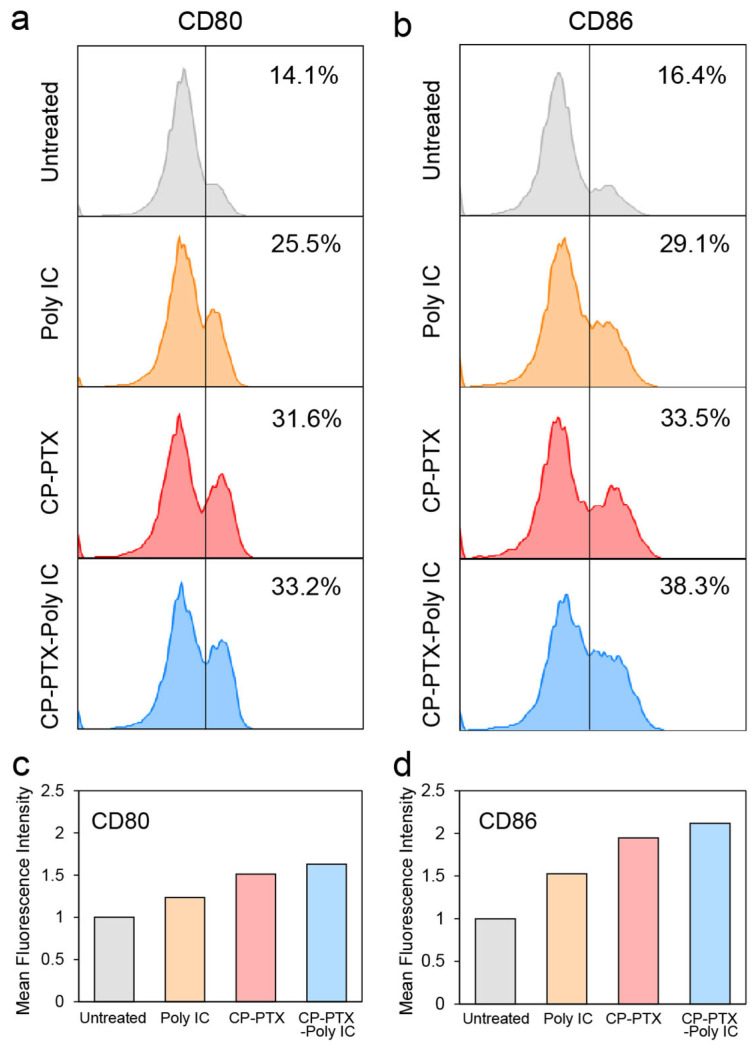
Flow cytometry study of DC maturation. (**a**) CD80 and (**b**) CD86 expressions in BMDCs were evaluated after treatment of Poly IC, CP-PTX, or CP-PTX-Poly IC for 24 h. The percentages of CD80 positive or CD86 positive populations are shown in each subfigure. The mean fluorescence intensity of (**c**) CD80 and (**d**) CD86 signals in BMDC were calculated from the treatment groups in (**a**,**b**). The dose of each treatment was the same amount of the corresponding components in CP-PTX-Poly IC at 10 μg/mL Poly IC (PTX = 850 μM).

The expression levels of CD80 and CD86, which are markers of BMDC maturation, were evaluated using flow cytometry. The results showed that treatment with Poly IC, CP-PTX, and CP-PTX-Poly IC elevated the expression levels of CD80 and CD86 in BMDCs compared to those found in untreated cells, as indicated by the percentage of CD80+ and CD86+ cells within the population of CD80 and CD86 (Figure 6a). Specifically, Poly IC alone triggered BMDC activation (25.5% of CD80+ and 29.1% of CD86+), while CP-PTX alone resulted in even greater BMDC activation (31.6% of CD80+ and 33.5% of CD86+). The combination treatment, CP-PTX-Poly IC, induced the highest levels of CD80 and CD86 expression (33.2% of CD80+ and 38.3% of CD86+), indicating the most significant activation of BMDCs. The mean fluorescence intensities (MFIs) of the CD80 and CD86 signals corroborated this finding. The MFIs of the CP-PTX-Poly IC-treated group exhibited values 1.6-fold (CD80) and 2.1-fold (CD86) higher than those of the untreated groups. These results indicated that CP-PTX-Poly IC effectively activated dendric cells via collective stimulation by Poly IC and PTX. In our previous report, the co-delivery of doxorubicin (DOX) and Poly IC was shown to enhance the killing of triple-negative breast cancer cells and the maturation of tumor-infiltrating dendritic cells. This study demonstrated that the co-delivery of DOX and Poly IC could effectively inhibit tumor growth and metastasis and extend survival in a mouse model, outperforming the efficacy of DOX alone [2]. Another clinical study explored the combination of cytotoxic chemotherapy agents (carboplatin and either paclitaxel or nanoparticle albumin-bound-paclitaxel) with PD-1/PD-L1 inhibitors (pembrolizumab) for non-small cell lung cancer (NSCLC) treatment. This combination showed improved median overall survival in patients compared to those treated with chemotherapy alone [41]. These studies highlight the potential of using combination therapies that include both chemotherapeutic and immunotherapeutic agents to improve cancer treatment outcomes in vivo.

### 3.7. Cytotoxicity of CP-PTX-Poly IC on BMDCs

**Figure 7:**
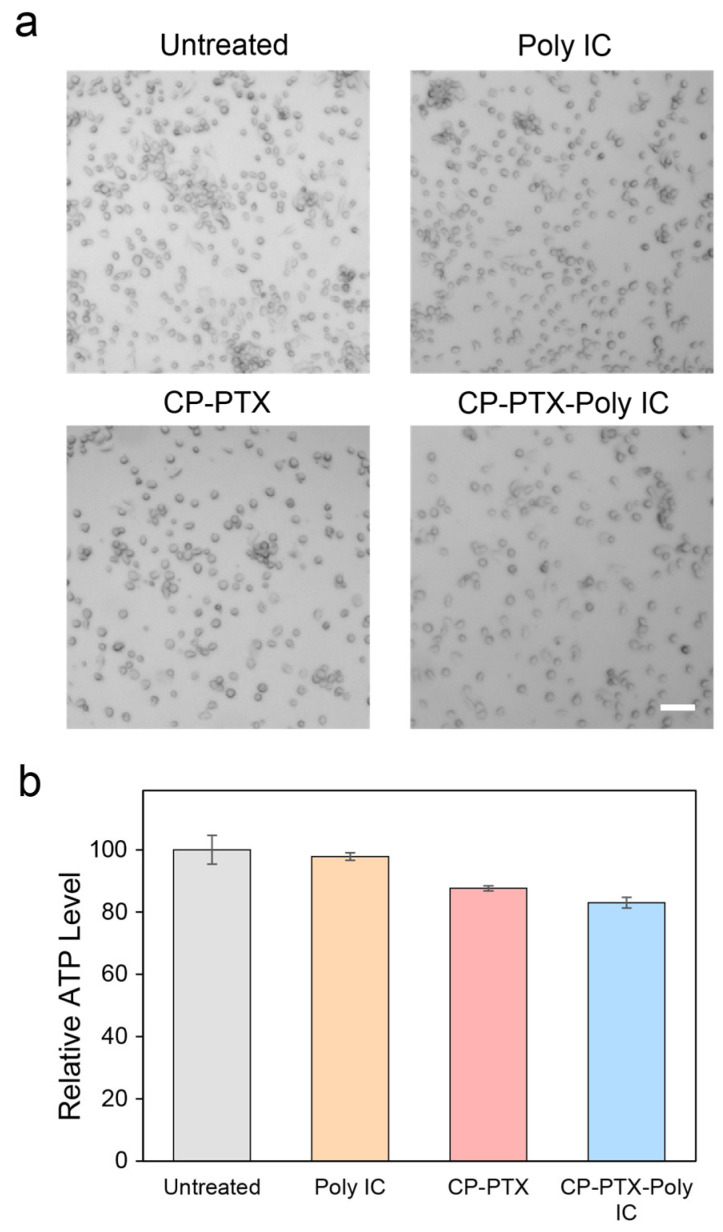
Biocompatibility of Poly IC, CP-PTX, and CP-PTX-Poly IC on BMDCs. (**a**) Brightfield images and (**b**) Relative ATP levels of untreated BMDCs or BMDCs treated by Poly IC, CP-PTX, and CP-PTX-Poly IC for 24 h. The dose of each treatment had the same amount of the corresponding components in CP-PTX-Poly IC at 10 μg/mL Poly IC (PTX = 850 μM). Scale bar is 50 μm.

While we expected that the CP-PTX-Poly IC agent would have a therapeutic effect on the target tumor cells, their potential cytotoxicity to non-target cells such as dendritic cells (DCs) is undesirable. Therefore, we tested the cytotoxicity of these agents against BMDCs (Figure 7).

BMDCs were treated with Poly IC, CP-PTX, and CP-PTX-Poly IC under the same treatment conditions as in the in vitro immune response study. Brightfield images were acquired to investigate cell morphology and numbers (Figure 7a). In all treatment groups, the BMDC cell morphology remained unchanged, and no cell enlargement due to the PTX effect was observed even when the PTX in PTX-containing groups was at high concentration of 850 μM. Although the cell numbers in the CP-PTX and CP-PTX-Poly IC group were slightly reduced, there were no apoptosis cells found in the images. To quantitatively assess cell viability, the ATP level in each treatment group was assessed with a CellTiter-Glo^®^ 3D cell viability assay kit. Compared to the ATP level of the untreated group, which was normalized at 100, the ATP levels of the CP-PTX and CP-PTX-Poly IC groups were slightly lower, at 87.6 and 83.0, respectively. The Poly IC group showed minimal changes in terms of ATP level at 97.8. In fact, dendritic cells have been reported to have a higher level of resistance to paclitaxel compared to tumor cells, probably due to their resistance to microtubule disruption, reduced cytokine production, and avoidance of apoptosis or necrosis at concentrations that are cytotoxic to tumor cells [42]. These results indicate that CP-PTX-Poly IC can effectively kill cancer cells through the action of PTX while simultaneously activating immune cells against cancer without causing significant toxicity to DCs.

These findings are important, as they demonstrate that CP-PTX-Poly IC has the dual capability of direct cancer cell cytotoxicity and immune system activation, making it a promising candidate for cancer combination therapy. The ability to inhibit tumor cells and stimulate an antitumor immune response without significant adverse effects on DCs suggests that CP-PTX-Poly IC nanoparticles could enhance therapeutic outcomes in cancer treatment.

## 4. Conclusions

In this study, we successfully developed an efficient chitosan-based nanoparticle fabrication approach for the co-delivery of a hydrophobic and hydrophilic drug combination, PTX and Poly IC, to exploit the synergistic effects of combined chemotherapy and immunotherapy. The CP-PTX-Poly IC nanoparticles were prepared through CP-drug conjugation followed by Poly IC and albumin incubations, resulting in small, homogenous, and stable nanoparticles. This innovative approach addresses the challenges of producing small, stable, and biocompatible nanoparticles for the effective co-delivery of drugs with distinct physicochemical properties.

Our findings demonstrated that the CP-PTX-Poly IC nanoparticles exhibited efficient cellular uptake across different cancer types, including breast cancer (4T1), liver cancer (HepG2), and pancreatic cancer (Pan02). The nanoparticles significantly reduced the viability of these cancer cells and enhanced dendritic cell (DC) maturation, which can lead to potent innate and adaptive antitumor immune responses in vivo. This dual-modal approach of co-delivering PTX and Poly IC presents a promising strategy for enhancing therapeutic outcomes in cancer treatment. The ability to inhibit tumor cells and activate the immune system simultaneously offers a comprehensive method of combating drug resistance and improving overall treatment efficacy. Our research demonstrates the potential of CP-PTX-Poly IC nanoparticles as a viable and effective option for advanced cancer therapy, paving the way for more potent and efficient treatments.

## Data Availability

Data can be available upon request from the authors.

## Supplementary Materials

The following supporting information can be downloaded at: https://www.mdpi.com/article/10.3390/pharmaceutics16101246/s1, Figure S1: Cumulative release of PTX from CP-PTX at pH 7.4 (blue line, PBS + 0.1% Triton X-100) and pH 5.4 (red line, sodium acetate buffer + 0.1% Triton X-100) at 37 °C; Figure S2: Physicochemical properties of CP-PTX-Poly IC nanoparticles. (a) Hydrodynamic size distri-bution of CP-PTX-poly IC with albumin tested in water. (b) Hydrodynamic size, PDI of CP-PTX-Poly IC tested in water and PBS and zeta potential and conductivity of CP-PTX-Poly IC tested in water.
