# Supplementary material for: An Efficient Fabrication Approach for Multi-Cancer Responsive Chemoimmuno Co-Delivery Nanoparticles
Source: Pharmaceutics. 2024 Sep 25;16(10):1246. doi: 10.3390/pharmaceutics16101246 (PMC11510515; doi:10.3390/pharmaceutics16101246)
Supplement: Supplementary file 1 [file pharmaceutics-16-01246-s001.zip › pharmaceutics-3167889-supplementary.pdf]

pharmaceutics

MDPI

Article

Supplementary Materials: An Efficient Fabrication Approach for Multi-Cancer Responsive Chemoimmuno Co-Delivery Nanoparticles

Jianxi Huang, Yu-Ting Chien, Qingxin Mu and Miqin Zhang

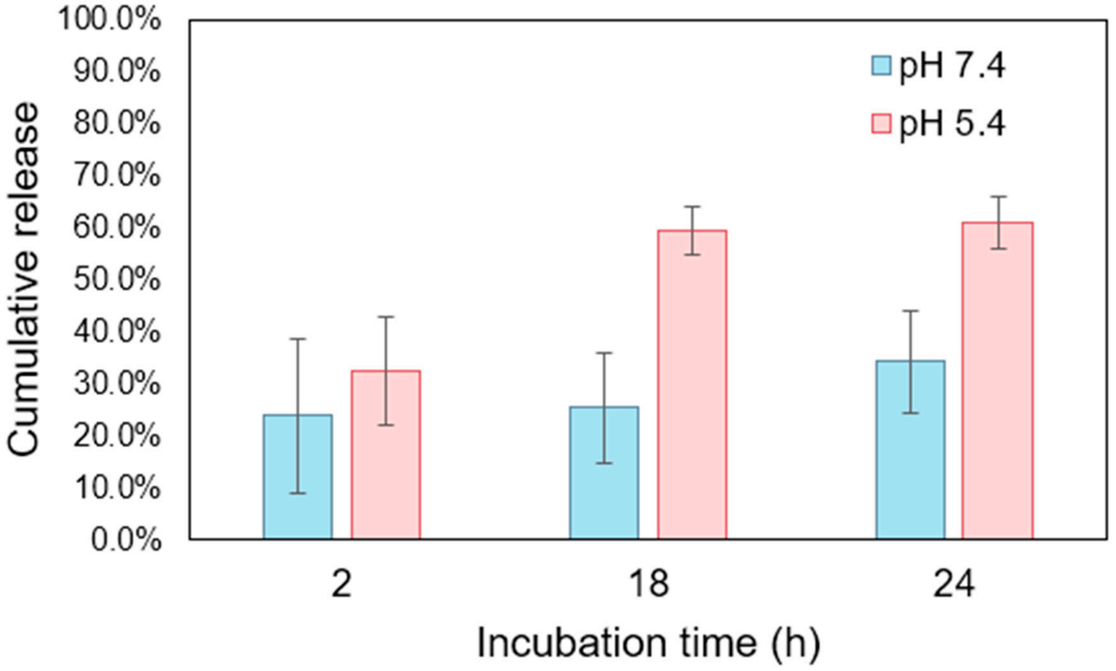

Figure S1. Cumulative release of PTX from CP-PTX at pH 7.4 (blue line, PBS + 0.1% Triton X-100) and pH 5.4 (red line, sodium acetate buffer + 0.1% Triton X-100) at 37 °C.

a

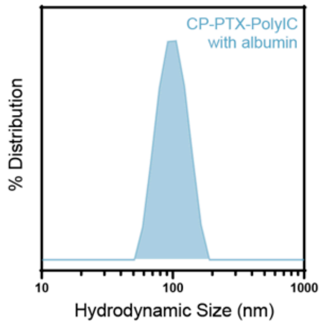

b

Sample	Hydrodynamic Size (nm)	PDI	Zeta Potential (mV)	Conductivity (mS/cm)
CP-PTX-Poly IC In water	108.3 ± 2.1	0.16 ± 0.0083	-12.2 ± 1.0	0.0093 ± 0.0012
CP-PTX-Poly IC In PBS	141.1 ± 28.3	0.34 ± 0.061	-	-

Figure S2. Physicochemical properties of CP-PTX-Poly IC nanoparticles. (a) Hydrodynamic size distribution of CP-PTX-poly IC with albumin tested in water. (b) Hydrodynamic size, PDI of CP-PTX-Poly IC tested in water and PBS and zeta potential and conductivity of CP-PTX-Poly IC tested in water.
